# Crystal structure, Hirshfeld surface analysis and computational study of a rhodamine B–salicyl­aldehyde Schiff base derivative

**DOI:** 10.1107/S2056989020007197

**Published:** 2020-06-05

**Authors:** Songwut Suramitr, Jitpinan Teanwarawat, Nuttapong Ithiapa, Worawat Wattanathana, Anwaraporn Suramitr

**Affiliations:** a Department of Chemistry, Faculty of Science and Center for Advanced Studies in Nanotechnology for Chemical, Food and Agricultural Industries, KU Institute for Advanced Studies, Kasetsart University, Bangkok 10900, Thailand; b Department of Materials Engineering, Faculty of Engineering, Kasetsart University 10900, Thailand; c Faculty of Science at Si Racha, Kasetsart University Si Racha Campus, Chonburi 20230, Thailand

**Keywords:** crystal structure, rhodamine B deriv­ative, Hirshfeld surface, computational study

## Abstract

The mol­ecular structure of the title compound {systematic name: 3′,6′-bis(di­ethyl­amino)-2-[(2-hy­droxy­benzyl­idene)amino]­spiro­[isoindoline-1,9′-xan­then]-3-one}, C_35_H_36_N_4_O_3_ or RbSa, can be seen as being composed of two parts sharing a central quaternary carbon atom. Both the xanthene and iso­indole moieties are nearly planar: 14 atoms in the former moiety show an r.m.s. deviation of 0.0411 Å and eleven atoms in the latter moiety show an r.m.s. deviation of 0.0545 Å. These two planes are almost perpendicular to each other, the angle between the mean planes being 87.71 (2)°. The title compound appears to be in its enol form. The corresponding H atom was located and freely refined at a distance of 1.02 (3) Å from the O atom and 1.72 (2) Å from the N atom. The strong intra­molecular hydrogen bond O—H⋯N bridging the hydroxyl group and its neighboring nitro­gen atom forms an *S*(6) graph-set motif. Apart from the intra­molecular O—H⋯N hydrogen bond, C—H⋯O inter­actions are observed between two neighbouring RbSa mol­ecules related by an inversion center. The C—O donor–acceptor distance is 3.474 (2) Å. Moreover, C—H⋯π inter­actions are observed between the C—H bond of one of the ethyl groups and the centroid of the benzene ring of the iso­indole moiety. The C⋯centroid distance is 3.8191 (15) Å. No π–π inter­actions are observed in the crystal structure as the shortest distance between ring centroids is more than 4 Å. A Hirshfeld surface analysis of the crystal structure indicates that the most important contributions for the crystal packing are from H⋯H, C⋯H/H⋯C, O⋯H/H⋯O and N⋯H/H⋯N inter­actions. DFT calculations at the CAM-B3LYP/6–31 G(*d*) level were carried out to gain a better understanding of the relative energies and the tautomerization process between two possible conformers (keto and enol), as well as the transition state of the title compound.

## Abstract 2

The mol­ecular structure of the title compound comprises two parts, xanthene and iso­indole, sharing a central quaternary carbon atom. Both the xanthene and iso­indole moieties are nearly planar and are almost perpendicular.

## Chemical context   

Rhodamine B derivatives are employed extensively as mol­ecular probes in the study of complex biological systems because of their high absorption coefficients, high fluorescence quantum yields and long-wavelength absorptions and emissions (Bao *et al.*, 2013 ▸; Biswal & Bag, 2013 ▸). On the basis of the spiro­lactam/ring-opened amide equilibrium of rhodamine, several fluorescence-based sensing systems for metal ions have been developed. Most of the reported sensors based on rhodamine B derivatives are fluorescent chemosensors for metal ion detection (Quang & Kim, 2010 ▸; Kim *et al.*, 2008 ▸; Kwon *et al.*, 2005 ▸; Liu *et al.*, 2013 ▸; Ni *et al.*, 2013 ▸). A rhodamine B derivative is usually used as a chemosensor because of its good photophysical properties, sensitivity, the low cost of chemical reagents and the ease of modifying its structure. Rhodamine B derivatives have naked-eye detection and show off–on fluorescent property when reacting with metal ions.
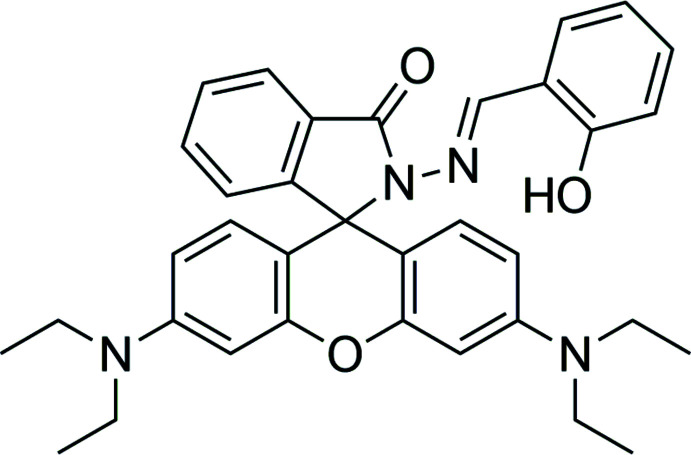


## Structural commentary   

**Figure 1:**
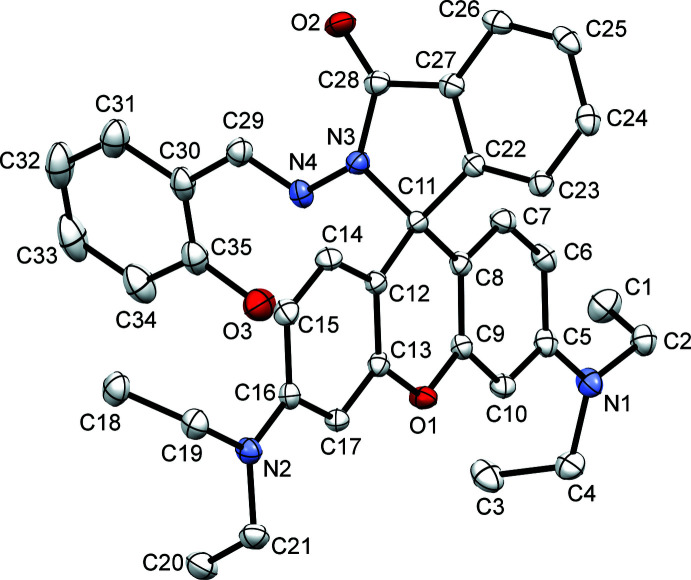
The mol­ecular structure of RbSa, showing the atom-labelling scheme. Displacement ellipsoids are drawn at the 50% probability level. The non-IUPAC atom labelling is for the convenience of discussion.

**Figure 2:**
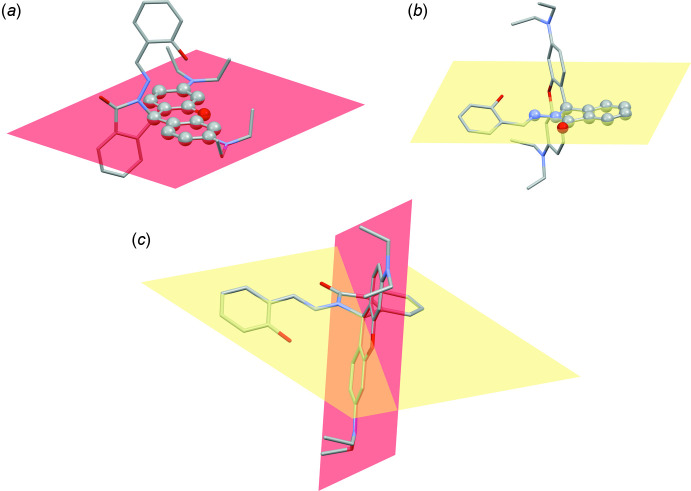
(*a*) The xanthene plane that is a mean plane constructed from 14 atoms, namely C5, C6–C10, O1, C11–C17. (*b*) a mean plane constructed from 11 atoms, namely C11, C22–C28, O2, N3, and N4. (*c*) View of the mol­ecule showing that planes (*a*) and (*b*) are roughly perpendicular to each other.

Fig. 1 ▸ shows the mol­ecular structure of the title compound (rhodamine B – salicyl­aldehyde derivative, RbSa) together with the atomic labelling scheme. The title compound crystallizes in the monoclinic space group *P*2_1_/c, and the asymmetric unit contains a single mol­ecule in a general position. The mol­ecule can be seen as having two distinct parts sharing a central quaternary carbon atom. The atoms in the xanthene moiety, namely C5–C10, O1, C11–C17, are almost coplanar, as seen from the r.m.s. deviation of 0.0411 Å (Fig. 2 ▸ *a*). The atoms C11, C22–C28, O2, N3 and N4 of the iso­indole unit are also nearly coplanar with an r.m.s. deviation of 0.0545 Å (Fig. 2 ▸ *b*). These two planes are almost perpendicular to each other, the dihedral angle between their mean planes being 87.71 (2)° (Fig. 2 ▸ *c*). The four ethyl groups present in the mol­ecule point out of the xanthene plane and are on the same side of the plane; the corresponding out-of-plane torsion angles C16—N2—C19—C18, C16—N2—C21—C20, C5—N1—C2—C1 and C5—N1—C4—C3 are 73.76 (15), −79.52 (15), −86.26 (15) and 65.09 (16)°, respectively.

**Figure 3:**
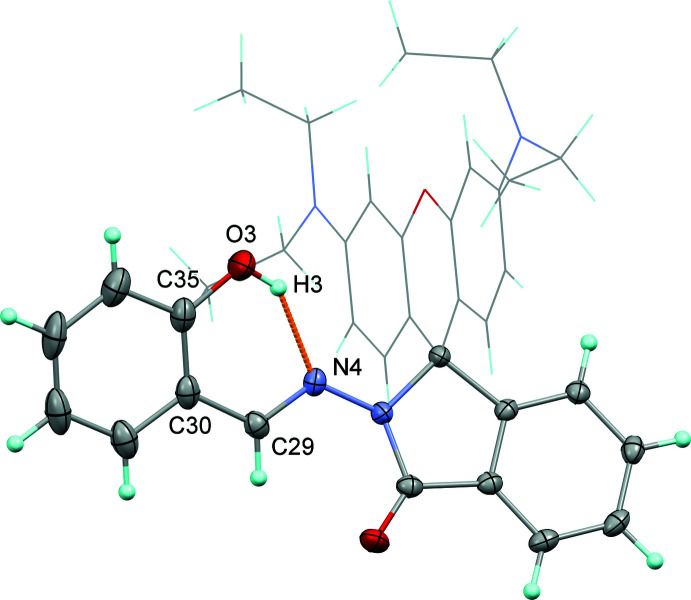
A view of mol­ecule illustrating the *S*(6) ring of the intra­molecular hydrogen bond constructed from the N4—C29—C30—C35—O3—H3 synthon.

The major tautomer of the rhodamine B – salicyl­aldehyde Schiff-base derivative is usually the enol form for compounds having hy­droxy and aza­methyl­ene groups attached to the benzene ring in the *ortho* positions (Veranitisagul *et al.*, 2012 ▸; Wattanathana *et al.*, 2012 ▸, 2016 ▸). Similarly, our title compound appears to be in its enol form. The corresponding H atom was located and freely refined at a distance of 1.02 (3) Å from the O atom and 1.72 (2) Å from the N atom (Table 1 ▸, Fig. 3 ▸). The strong intra­molecular O—H⋯N hydrogen bond bridging the hydroxyl group and its neighbouring nitro­gen atom forms an *S*(6) graph-set motif involving atoms N4, H3, O3, C35, C30 and C29 and stabilizes a rigid configuration that can partially inhibit the rotation of the phenyl ring about the N—N bond.

## Supra­molecular features   

**Figure 4:**
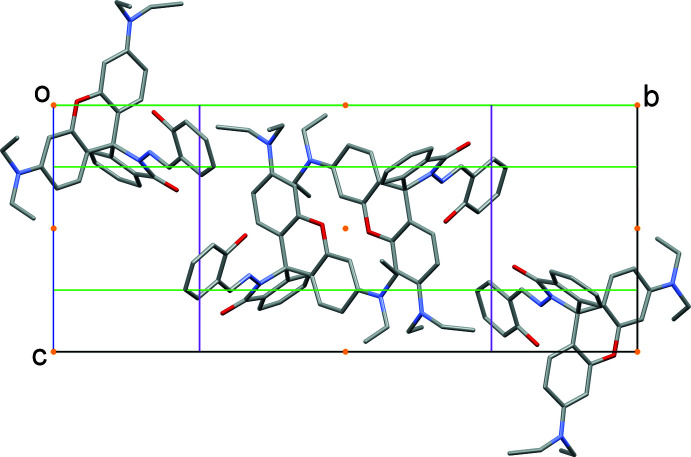
The unit cell and mol­ecular packing in the title compound with the unit-cell contents projecting down the [100] direction. The orange dots represent inversion centres and the green and magenta lines represent glide planes and twofold axes, respectively.

**Figure 5:**
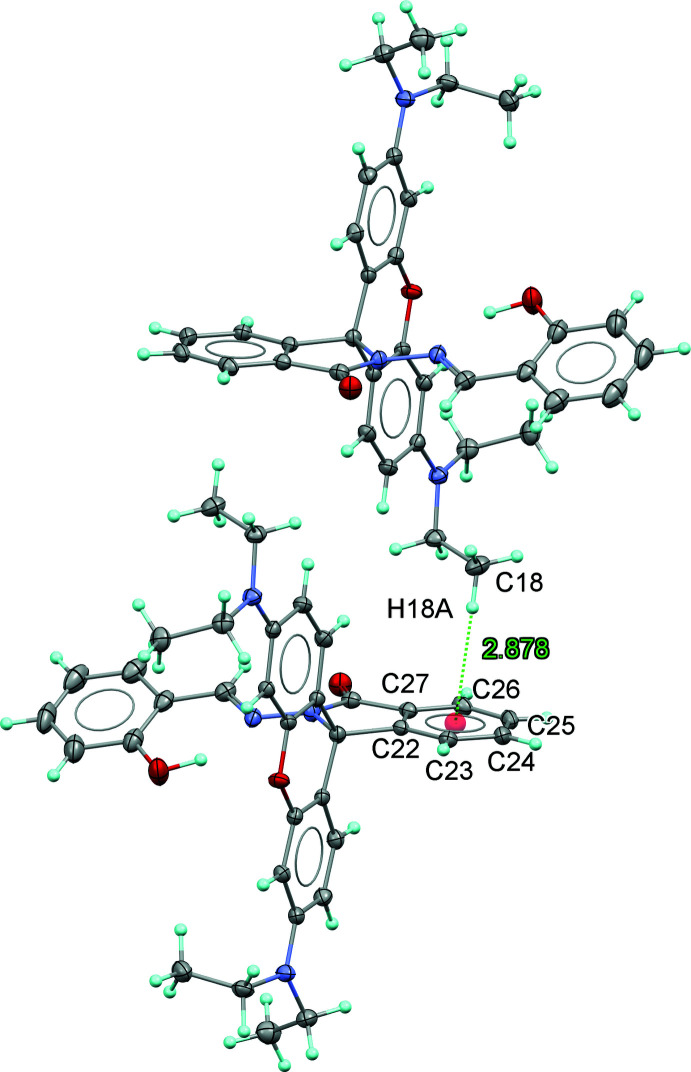
A view of the crystal packing showing C—H⋯π inter­actions between C18—H18*A* and the C22–C27 ring of the iso­indole unit.

Besides the intra­molecular O—H⋯N hydrogen bond, the title mol­ecules form C31–H31⋯O3^i^ inter­actions [symmetry code: (i) *x*, −*y* + 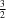, *z* − 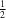] between two neighbouring RbSa mol­ecules related by an inversion center (Fig. 4 ▸). The C31⋯O3^i^ donor-acceptor distance is 3.474 (2) Å. Moreover, C—H⋯π inter­actions are observed between C18—H18*A* and the benzene ring (C22–C27) of the iso­indole unit (Fig. 5 ▸). Within the crystal structure, the RbSa mol­ecules aggregate into infinite mol­ecular chains in an end-to-end packing mode along the [100] direction. No π–π inter­actions are observed in the crystal structure as the shortest distance between ring centroids is greater than 4 Å.

## Hirshfeld surface analysis   

**Figure 6:**
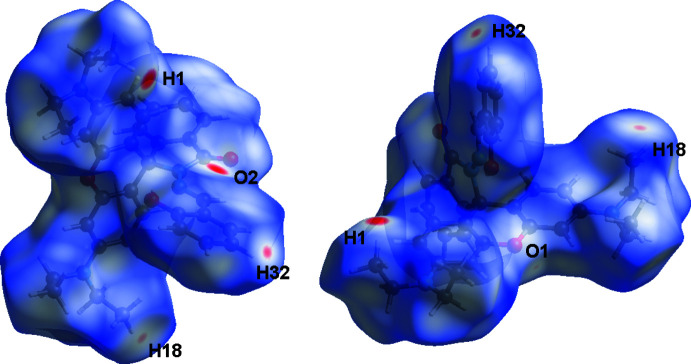
Two views of the three-dimensional Hirshfeld surface of the title compound plotted over *d*_norm_ in the range −0.1732 to 1.4064 a.u.

**Figure 7:**
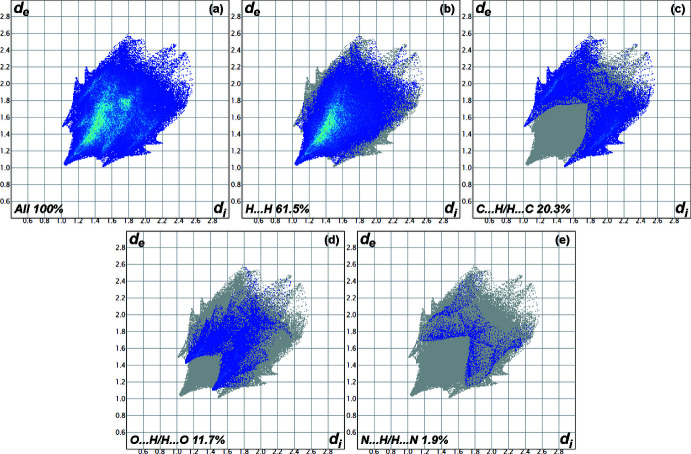
The full two-dimensional fingerprint plots for the title compound, showing (*a*) all inter­actions and those delineated into (*b*) H⋯H, (*c*) C⋯H/H⋯C, (*d*) O⋯H/H⋯O, and (*e*) N⋯H/H⋯N inter­actions.

The inter­molecular inter­actions in the crystal of the title compound were investigated and visualized by performing a Hirshfeld surface (HS) analysis (Hirshfeld, 1977 ▸; Spackman & Jayatilaka, 2009 ▸) using *Crystal Explorer 17.5* software (Turner *et al.*, 2017 ▸). The HS plotted over *d*_norm_ in the range −0.1732 to 1.4064 a.u. is shown in Fig. 6 ▸. Fig. 7 ▸ shows the full two-dimensional fingerprint plot (McKinnon *et al.*, 2007 ▸) and those delineated into the major contacts: H⋯H (61.5%), H⋯C/C⋯H (20.3%), H⋯O/O⋯H (11.7%), and H⋯N/N⋯H (1.9%) for which *d*_e_ + *d*_i_ ∼2.0, 3.0, 2.8 and 3.4 Å, respectively. The other contacts are negligible with individual contributions of less than 1% and a sum of less than 5%.

## Database survey   

A search of the Cambridge Structural Database (CSD version 5.41, November 2019 + one update; Groom *et al.*, 2016 ▸) shows that the crystal structures of many compounds having rhodamine B as a core have been reported. The structural diversity of rhodamine B derivatives results from the fact that the different functional groups in the mol­ecules can be tuned. For example, the carb­oxy­lic functional group can be converted to ester functional groups such as methyl ester derivatives (I) (ROKNOU; Fun *et al.*, 1997 ▸) and (II) (QIMMII; Adhikesavalu *et al.*, 2001 ▸), ethyl ester derivatives (III) (QIMMEE; Adhikesavalu *et al.*, 2001 ▸) and cyclic lactones (IV) (FUFTIJ; Kvick *et al.*, 2000 ▸). In our case, the carb­oxy­lic acid functional group is sequentially transformed into a cyclic lactam with a hydrazone side chain. The counter-anions are the other key factor in the crystal structures of rhodamine B derivatives. Mizuguchi (2008 ▸) reported the crystal structure of the ethyl gallate salt at 93 K (PIHJIA01), and Venkatraman *et al.* (2008 ▸) published a new derivative with the hexa­chlorido­stannate(IV) anion, RISQIU. Moreover, rhodamine B derivatives can be used as a ligand for many metal cations to form coordination complexes such as the cadmium complex (V) (IKISUQ; Qu *et al.*, 2001 ▸).

## Synthesis and crystallization   

The reagents were purchased from commercial suppliers and used without further purification: rhodamine B (Fluka Chemicals), hydrazine hydrate (Across Organics), salicyaldehyde (Sigma–Aldrich), hydro­chloric acid (Valchem), ammonium hydroxide (Mallinckrodt Chemicals) and sodium hydroxide (RCI Labscan). Solvents including absolute ethanol (EtOH), methanol (MeOH) and chloroform were purchased from RCI Labscan and Merck. ^1^H NMR spectra were measured on a Varian INOVA 400 spectrometer at 400 MHz in CDCl_3_. The FTIR spectrum was obtained using a Bruker Tensor 27 spectrometer, while the mass spectrum was recorded on a Bruker microTOF-Q III. The melting point of the obtained sample was measured by a Stuart Scientific melting-point analyser (SMP10).

**Figure 8:**
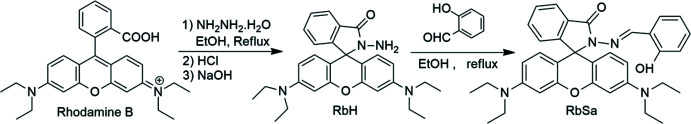
The synthetic route and the structures of RbSa.

Fig. 8 ▸ shows the chemical structures of the starting materials and inter­mediate and the synthetic route of the rhodamine–Schiff-base derivative studied in this work (RbSa). Firstly, the RbH inter­mediate compound was synthesized from the reaction between rhodamine B and hydrazine hydrate. Rhodamine B (1.20 g, 2.50 mmol) was dissolved in ethanol. An equimolar amount of hydrazine hydrate (2.0 ml, 2.5 mmol) was added to this solution. The mixture was then refluxed at 373 K under N_2_ for 2 h. During the reaction, the colour of the solution changed from dark purple to light orange. After the reaction was complete, a precipitate of RbH was obtained by solvent evaporation, and then 1 *M* HCl was added to re-dissolve the crude product to obtain a clear red solution. After that, 1 *M* NaOH was added slowly in order to precipitate the purer RbH compound out. The obtained RbH was filtered under reduced pressure. The title compound RbSa was then prepared from the reaction of the filtered RbH (0.48 g, 0.97 mmol) and salicyl­aldehyde (0.12 ml, 1 mmol) in ethano­lic solution. A pink precipitate of RbSa formed after reflux at 353 K for 12 h under an N_2_ atmosphere. The RbSa precipitate was separated by vacuum filtration and washed three times with cold ethanol. After recrystallization from chloro­form and methanol mixed solvents with a volume ratio of 1:1, light-violet single crystals were obtained after several days, m.p. 495 K.

HR–MS (ESI–TOF) *m*/*z*: [*M* + 1]^+^ calculated from C_35_H_37_N_4_O_3_ is 561.286017; found 561.288140.

^1^H NMR (400 MHz, CDCl_3_) chemical shifts (δ): 9.31 (*s*, 1H), 8.10–7.90 (*m*, 1H), 7.56 (*s*, 2H), 7.18 (*dq*, *J* = 7.2, 4.3, 3.9 Hz, 2H), 7.13 (*d*, *J* = 7.8 Hz, 1H), 6.81 (*q*, *J* = 7.9, 7.5 Hz, 3H), 6.41 (*d*, *J* = 87.7 Hz, 5H), 3.33 (*s*, 8H), 1.18 (*s*, 12H).

## Computational details   

All reported calculations (geometry optimizations, vibrational frequencies, and relative energies) were performed using *Gaussian09* (Frisch *et al.*, 2009 ▸) using density functional theory (DFT) at the CAM-B3LYP/6–31 G(*d*) level. The ground-state geometries of the RbSa mol­ecules with different conformers were optimized, and vibrational frequency calculations were performed to confirm that the optimized structures correspond well to a local minimum or a transition state. The hybrid exchange-correlation functional CAM-B3LYP was selected because it has been found to be a method of choice in important reported studies (Klinhom *et al.*, 2019 ▸; Miengmern *et al.*, 2019 ▸), showing a good compromise between computational time and the accuracy of the results. All calculations were carried out in ethanol using a conductor-like polarizable continuum model (CPCM) (Scalmani *et al.*, 2006 ▸).

## Quantum chemical calculations   

**Figure 9:**
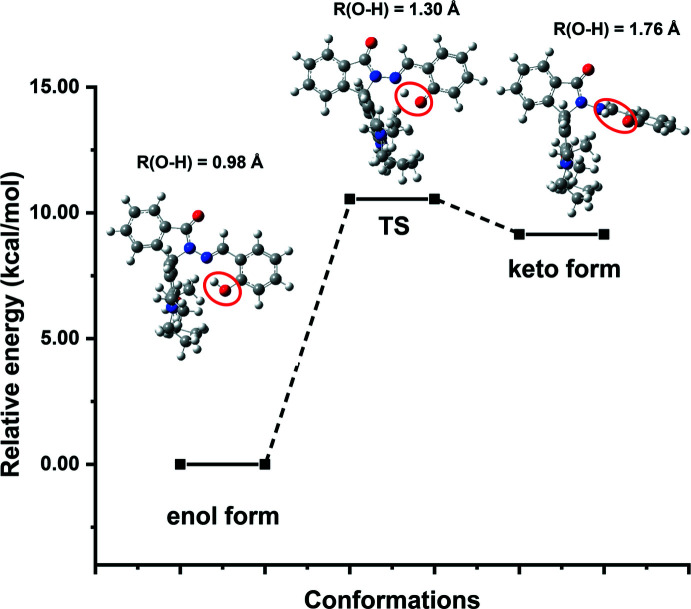
Optimized structures and relative energies of the enol form, the keto form and the transition state formed during the tautomerization process of the compound RbSa, calculated in ethanol at the CAM-B3LYP level using a 6–311 G(d,p) basis set.

For a more in-depth insight into the molecular structures of RbSa, density functional theory (DFT) calculations at the CAM-B3LYP/6-311 G(d,p) level were carried out. They were mainly applied to investigate the intra­molecular proton-transfer reaction during the enol–keto tautomerization mechanism. The mol­ecular structures of two essential conformers (the enol and keto forms) of the title compound were first obtained by geometry optimizations without any constraints. Starting from the enol form and going to the keto form, the potential energy curve (PEC) was explored by elongating the O—H bond in steps of 0.1 Å (from 1.0 to 1.8 Å). The CAM-B3LYP optimized conformers and their relative energies are depicted in Fig. 9 ▸.

The enol form is the most stable conformer (O—H = 0.98 Å) with the lowest relative energy, and the keto form (O—H = 1.76 Å) is found to be slightly higher in energy than the enol form by 9.1 kcal mol^−1^. All along the tautomerization path, the six atoms involved in the initial *S*(6) graph-set motif remain coplanar, but a rotation occurs around the N—N bond between the di­aminoxanthene and the salicyl­idene aniline moieties, which move from being coplanar in the enol form to being nearly perpendicular in the keto-form (Fig. 9 ▸). The rotation of the N—N bond gives rise to the elongation and then the breakage of the O—H bond in the enol form, resulting in the formation of the N—H bond (keto form). Although the enol–keto tautomerization involves breakage of the O—H bond, the intra­molecular hydrogen-bonded *S*(6) ring still remains even in the keto form, involving the same atoms in the synthon as in the enol form. According to the relative energy calculations, it can be concluded that the phenolic –OH group can form a stronger intra­molecular hydrogen-bonding inter­action (enol form) with the N atom of the di­aminoxanthene moiety than with the –NH group of the phenolate O in the keto closed form. The result is in a good agreement with the X-ray crystal structure in that the enol form is the dominant conformer.

Based on the highest energy structure obtained on the PEC, we optimized the mol­ecular geometry of the transition state of the keto–enol tautomerization pathway without constraints. The single negative (imaginary) vibrational frequency calculated for the obtained structure showed that the transition state was correctly determined. The transition state lies 10.4 kcal mol^−1^ higher in energy than the enol form and only 1.3 kcal mol^−1^ above that of the keto form (Fig. 9 ▸). The optimized structure reveals an O—H distance of 1.30 Å, in between the O—H distances observed in the enol and keto conformers, but closer to that of the enol-conformer. The energy curve is rather flat between the transition state and the keto form, despite a difference of about 0.5 Å between the O—H distances (1.30 and 1.76 Å, respectively). Besides the fact that the enol form exhibits the lowest absolute energy and is the most stable conformer, that flatness also explains why the keto form is not as stable and can easily return to the enol form in solution under normal conditions.

## Refinement   

Crystal data, data collection and structure refinement details are summarized in Table 2 ▸. The O-bound H atom (H3) was located in a difference-Fourier map and freely refined. The other hydrogen atoms were refined using a riding model with *d*(C—H) = 0.95 Å and *U*_iso_(H) = 1.2*U*_eq_(C) for aromatic hydrogen atoms, with *d*(C—H) = 0.99 Å and *U*_iso_(H) = 1.2*U*_eq_(C) for –CH_2_– hydrogen atoms and *d*(C–H) = 0.98 Å, *U*_iso_(H) = 1.5*U*_eq_(C) for the terminal methyl hydrogen atoms.

## Supplementary Material

Crystal structure: contains datablock(s) I. DOI: 10.1107/S2056989020007197/zq2251sup1.cif

Structure factors: contains datablock(s) I. DOI: 10.1107/S2056989020007197/zq2251Isup2.hkl

Supporting information file. DOI: 10.1107/S2056989020007197/zq2251Isup3.cml

CCDC reference: 2006371

Additional supporting information:  crystallographic information; 3D view; checkCIF report

## Figures and Tables

**Table 1:** Hydrogen-bond geometry (Å, °) *Cg* is the centroid of the C22–C27 ring.

*D*—H⋯*A*	*D*—H	H⋯*A*	*D*⋯*A*	*D*—H⋯*A*
C31—H31⋯O3^i^	0.95	2.77	3.4737 (19)	131
O3—H3⋯N4	1.02 (3)	1.72 (2)	2.6276 (15)	145 (2)
C18—H18*A*⋯*Cg* ^ii^	0.98	2.88	3.8191 (15)	161

Symmetry codes: (i) 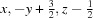; (ii) 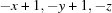.

**Table 2:** Experimental details

Crystal data	
Chemical formula	C_35_H_36_N_4_O_3_
*M*_r_	560.68
Crystal system, space group	Monoclinic, *P*2_1_/*c*
Temperature (K)	100
*a*, *b*, *c* (Å)	9.3903 (7), 26.8178 (19), 11.5639 (8)
β (°)	101.635 (2)
*V* (Å^3^)	2852.3 (4)
*Z*	4
Radiation type	Cu *K*α
μ (mm^−1^)	0.67
Crystal size (mm)	0.20 × 0.20 × 0.20

Data collection	
Diffractometer	Bruker APEXIII CCD
Absorption correction	Multi-scan (*SADABS*; Bruker, 2016 ▸)
*T*_min_, *T*_max_	0.673, 0.754
No. of measured, independent and observed [*I* > 2σ(*I*)] reflections	64500, 5508, 5462
*R*_int_	0.030
(sin θ/λ)_max_ (Å^−1^)	0.618

Refinement	
*R*[*F* ^2^ > 2σ(*F* ^2^)], *wR*(*F* ^2^), *S*	0.042, 0.099, 1.07
No. of reflections	5508
No. of parameters	387
H-atom treatment	H atoms treated by a mixture of independent and constrained refinement
Δρ_max_, Δρ_min_ (e Å^−3^)	0.26, −0.25

Computer programs: *APEX3* and *SAINT* (Bruker, 2018 ▸), *SHELXT* (Sheldrick, 2015*a* ▸), *SHELXL* (Sheldrick, 2015*b* ▸), *OLEX2* (Dolomanov *et al.*, 2009 ▸) and *Mercury* (Macrae *et al.*, 2020 ▸).

## supplementary crystallographic information

### Crystal data

**Table:**

C_35_H_36_N_4_O_3_	*F*(000) = 1192
*M**_r_* = 560.68	*D*_x_ = 1.306 Mg m^−^^3^
Monoclinic, *P*2_1_/*c*	Cu *K*α radiation, λ = 1.54178 Å
*a* = 9.3903 (7) Å	Cell parameters from 9681 reflections
*b* = 26.8178 (19) Å	θ = 3.9–72.1°
*c* = 11.5639 (8) Å	µ = 0.67 mm^−^^1^
β = 101.635 (2)°	*T* = 100 K
*V* = 2852.3 (4) Å^3^	Block, clear light violet
*Z* = 4	0.20 × 0.20 × 0.20 mm

### Data collection

**Table:**

Bruker APEXIII CCD diffractometer	5462 reflections with *I* > 2σ(*I*)
φ and ω scans	*R*_int_ = 0.030
Absorption correction: multi-scan (SADABS; Bruker, 2016)	θ_max_ = 72.2°, θ_min_ = 5.1°
*T*_min_ = 0.673, *T*_max_ = 0.754	*h* = −11→11
64500 measured reflections	*k* = −33→31
5508 independent reflections	*l* = −14→14

### Refinement

**Table:**

Refinement on *F*^2^	Primary atom site location: dual
Least-squares matrix: full	Hydrogen site location: mixed
*R*[*F*^2^ > 2σ(*F*^2^)] = 0.042	H atoms treated by a mixture of independent and constrained refinement
*wR*(*F*^2^) = 0.099	*w* = 1/[σ^2^(*F*_o_^2^) + (0.0366*P*)^2^ + 1.6105*P*] where *P* = (*F*_o_^2^ + 2*F*_c_^2^)/3
*S* = 1.07	(Δ/σ)_max_ = 0.001
5508 reflections	Δρ_max_ = 0.26 e Å^−^^3^
387 parameters	Δρ_min_ = −0.25 e Å^−^^3^
0 restraints	

### Special details

**Table:**

Geometry. All esds (except the esd in the dihedral angle between two l.s. planes) are estimated using the full covariance matrix. The cell esds are taken into account individually in the estimation of esds in distances, angles and torsion angles; correlations between esds in cell parameters are only used when they are defined by crystal symmetry. An approximate (isotropic) treatment of cell esds is used for estimating esds involving l.s. planes.

### Fractional atomic coordinates and isotropic or equivalent isotropic displacement parameters (Å^2^)

**Table:**

	*x*	*y*	*z*	*U*_iso_*/*U*_eq_	
O1	0.63421 (9)	0.53998 (3)	0.51482 (7)	0.01990 (19)	
O2	0.35333 (10)	0.71057 (3)	0.15605 (8)	0.0258 (2)	
O3	0.85258 (11)	0.67163 (4)	0.47128 (9)	0.0341 (2)	
N3	0.47258 (11)	0.64737 (4)	0.27590 (9)	0.0182 (2)	
N2	0.78424 (11)	0.43998 (4)	0.22361 (9)	0.0203 (2)	
N4	0.61281 (11)	0.66475 (4)	0.30950 (9)	0.0197 (2)	
N1	0.54337 (12)	0.63079 (4)	0.84325 (9)	0.0228 (2)	
C27	0.22734 (13)	0.63956 (4)	0.22148 (10)	0.0181 (2)	
C12	0.52489 (12)	0.55798 (4)	0.31027 (10)	0.0163 (2)	
C9	0.56210 (12)	0.58015 (4)	0.55053 (10)	0.0167 (2)	
C22	0.27539 (12)	0.59856 (4)	0.29156 (10)	0.0165 (2)	
C8	0.46927 (12)	0.61076 (4)	0.47253 (10)	0.0165 (2)	
C13	0.61868 (12)	0.53104 (4)	0.39588 (10)	0.0163 (2)	
C17	0.70486 (13)	0.49250 (4)	0.36853 (10)	0.0174 (2)	
H17	0.767903	0.475358	0.430372	0.021*	
C28	0.35267 (13)	0.67139 (4)	0.21032 (10)	0.0185 (2)	
C16	0.70031 (12)	0.47851 (4)	0.25124 (10)	0.0173 (2)	
C10	0.58838 (13)	0.58696 (5)	0.67180 (10)	0.0188 (2)	
H10	0.652938	0.565077	0.721574	0.023*	
C11	0.43818 (12)	0.60188 (4)	0.34033 (10)	0.0165 (2)	
C7	0.40438 (13)	0.64983 (5)	0.52325 (11)	0.0199 (3)	
H7	0.340634	0.671699	0.472730	0.024*	
C5	0.52106 (13)	0.62564 (5)	0.72189 (11)	0.0190 (2)	
C6	0.42897 (13)	0.65798 (5)	0.64345 (11)	0.0212 (3)	
H6	0.383917	0.685408	0.673746	0.025*	
C24	0.03086 (13)	0.57033 (5)	0.26884 (11)	0.0219 (3)	
H24	−0.038019	0.546594	0.284338	0.026*	
C23	0.17839 (13)	0.56257 (5)	0.31441 (10)	0.0190 (2)	
H23	0.211332	0.533686	0.359496	0.023*	
C15	0.60465 (13)	0.50567 (5)	0.16302 (11)	0.0207 (3)	
H15	0.598389	0.497589	0.082170	0.025*	
C26	0.08054 (14)	0.64726 (5)	0.17477 (11)	0.0219 (3)	
H26	0.048406	0.675417	0.126699	0.026*	
C14	0.52070 (13)	0.54367 (5)	0.19346 (11)	0.0203 (3)	
H14	0.457022	0.560945	0.132251	0.024*	
C25	−0.01736 (13)	0.61228 (5)	0.20097 (11)	0.0231 (3)	
H25	−0.118581	0.617015	0.172246	0.028*	
C29	0.66197 (14)	0.69802 (5)	0.24752 (11)	0.0226 (3)	
H29	0.603743	0.709238	0.175314	0.027*	
C21	0.88015 (14)	0.41198 (5)	0.31614 (11)	0.0228 (3)	
H21A	0.901201	0.379277	0.283556	0.027*	
H21B	0.827910	0.405544	0.380933	0.027*	
C30	0.80710 (15)	0.71842 (5)	0.28840 (12)	0.0249 (3)	
C4	0.67041 (14)	0.60808 (5)	0.91783 (11)	0.0250 (3)	
H4A	0.670275	0.571911	0.900719	0.030*	
H4B	0.663083	0.612091	1.001535	0.030*	
C19	0.78644 (15)	0.42836 (5)	0.10088 (11)	0.0230 (3)	
H19A	0.685046	0.427015	0.055715	0.028*	
H19B	0.829533	0.394843	0.097343	0.028*	
C2	0.46026 (14)	0.66609 (5)	0.89867 (11)	0.0236 (3)	
H2A	0.454398	0.653305	0.977887	0.028*	
H2B	0.359964	0.667543	0.851350	0.028*	
C35	0.89543 (15)	0.70507 (5)	0.39724 (13)	0.0284 (3)	
C3	0.81405 (15)	0.63038 (6)	0.90053 (12)	0.0310 (3)	
H3A	0.820426	0.627884	0.817176	0.046*	
H3B	0.894997	0.612070	0.948926	0.046*	
H3C	0.819047	0.665505	0.924369	0.046*	
C18	0.87120 (16)	0.46553 (5)	0.04151 (12)	0.0287 (3)	
H18A	0.866719	0.455502	−0.040637	0.043*	
H18B	0.972824	0.466195	0.083581	0.043*	
H18C	0.828601	0.498805	0.043386	0.043*	
C20	1.02371 (15)	0.43748 (5)	0.36756 (13)	0.0291 (3)	
H20A	1.077556	0.443530	0.304608	0.044*	
H20B	1.081375	0.416020	0.428003	0.044*	
H20C	1.004643	0.469316	0.403179	0.044*	
C1	0.52129 (17)	0.71867 (5)	0.91209 (13)	0.0319 (3)	
H1A	0.517760	0.733206	0.833812	0.048*	
H1B	0.622293	0.717668	0.955531	0.048*	
H1C	0.463219	0.739067	0.955591	0.048*	
C31	0.85986 (17)	0.75357 (5)	0.21765 (14)	0.0332 (3)	
H31	0.800983	0.763165	0.144281	0.040*	
C32	0.99603 (19)	0.77443 (6)	0.25302 (17)	0.0412 (4)	
H32	1.031113	0.797800	0.203696	0.049*	
C34	1.03266 (16)	0.72693 (6)	0.43299 (16)	0.0377 (4)	
H34	1.092100	0.718219	0.506806	0.045*	
C33	1.08133 (17)	0.76114 (6)	0.36078 (17)	0.0430 (4)	
H33	1.174654	0.775796	0.385279	0.052*	
H3	0.752 (3)	0.6591 (9)	0.431 (2)	0.069 (7)*	

### Atomic displacement parameters (Å^2^)

**Table:**

	*U*^11^	*U*^22^	*U*^33^	*U*^12^	*U*^13^	*U*^23^
O1	0.0224 (4)	0.0236 (4)	0.0129 (4)	0.0070 (3)	0.0017 (3)	−0.0005 (3)
O2	0.0281 (5)	0.0213 (5)	0.0272 (5)	0.0023 (4)	0.0035 (4)	0.0077 (4)
O3	0.0246 (5)	0.0390 (6)	0.0363 (6)	−0.0069 (4)	0.0001 (4)	0.0078 (4)
N3	0.0162 (5)	0.0190 (5)	0.0188 (5)	−0.0012 (4)	0.0022 (4)	0.0035 (4)
N2	0.0205 (5)	0.0219 (5)	0.0190 (5)	0.0026 (4)	0.0049 (4)	−0.0014 (4)
N4	0.0169 (5)	0.0197 (5)	0.0233 (5)	−0.0016 (4)	0.0062 (4)	−0.0019 (4)
N1	0.0207 (5)	0.0305 (6)	0.0171 (5)	0.0025 (4)	0.0034 (4)	−0.0042 (4)
C27	0.0193 (6)	0.0197 (6)	0.0148 (5)	0.0016 (5)	0.0025 (4)	−0.0018 (4)
C12	0.0140 (5)	0.0188 (6)	0.0162 (5)	−0.0012 (4)	0.0028 (4)	0.0014 (4)
C9	0.0137 (5)	0.0185 (6)	0.0180 (6)	−0.0007 (4)	0.0036 (4)	−0.0005 (4)
C22	0.0154 (6)	0.0204 (6)	0.0134 (5)	0.0012 (4)	0.0023 (4)	−0.0022 (4)
C8	0.0134 (5)	0.0195 (6)	0.0164 (6)	−0.0024 (4)	0.0024 (4)	0.0003 (4)
C13	0.0154 (5)	0.0200 (6)	0.0134 (5)	−0.0023 (4)	0.0030 (4)	0.0003 (4)
C17	0.0155 (5)	0.0206 (6)	0.0157 (5)	0.0006 (4)	0.0020 (4)	0.0026 (4)
C28	0.0211 (6)	0.0188 (6)	0.0155 (5)	0.0021 (5)	0.0031 (4)	−0.0002 (4)
C16	0.0152 (5)	0.0185 (6)	0.0190 (6)	−0.0023 (4)	0.0053 (4)	−0.0004 (4)
C10	0.0162 (5)	0.0230 (6)	0.0164 (6)	0.0005 (5)	0.0014 (4)	0.0006 (5)
C11	0.0151 (5)	0.0177 (6)	0.0163 (6)	−0.0009 (4)	0.0022 (4)	0.0028 (4)
C7	0.0173 (6)	0.0210 (6)	0.0206 (6)	0.0011 (5)	0.0017 (5)	0.0008 (5)
C5	0.0152 (5)	0.0238 (6)	0.0178 (6)	−0.0035 (5)	0.0030 (4)	−0.0027 (5)
C6	0.0187 (6)	0.0220 (6)	0.0230 (6)	0.0005 (5)	0.0049 (5)	−0.0042 (5)
C24	0.0182 (6)	0.0286 (7)	0.0190 (6)	−0.0046 (5)	0.0043 (5)	−0.0040 (5)
C23	0.0190 (6)	0.0223 (6)	0.0155 (5)	−0.0009 (5)	0.0031 (4)	0.0000 (4)
C15	0.0204 (6)	0.0270 (6)	0.0146 (6)	0.0005 (5)	0.0030 (5)	−0.0011 (5)
C26	0.0207 (6)	0.0237 (6)	0.0193 (6)	0.0055 (5)	−0.0010 (5)	−0.0020 (5)
C14	0.0184 (6)	0.0260 (6)	0.0152 (6)	0.0022 (5)	0.0004 (4)	0.0030 (5)
C25	0.0154 (6)	0.0310 (7)	0.0211 (6)	0.0033 (5)	−0.0005 (5)	−0.0059 (5)
C29	0.0251 (6)	0.0210 (6)	0.0233 (6)	0.0005 (5)	0.0089 (5)	0.0002 (5)
C21	0.0251 (6)	0.0182 (6)	0.0251 (6)	0.0039 (5)	0.0051 (5)	0.0008 (5)
C30	0.0247 (6)	0.0198 (6)	0.0337 (7)	−0.0014 (5)	0.0140 (5)	−0.0049 (5)
C4	0.0279 (7)	0.0312 (7)	0.0150 (6)	0.0024 (5)	0.0024 (5)	−0.0011 (5)
C19	0.0266 (6)	0.0218 (6)	0.0216 (6)	0.0006 (5)	0.0070 (5)	−0.0046 (5)
C2	0.0243 (6)	0.0268 (7)	0.0209 (6)	−0.0003 (5)	0.0077 (5)	−0.0025 (5)
C35	0.0245 (7)	0.0244 (7)	0.0389 (8)	−0.0034 (5)	0.0127 (6)	−0.0033 (6)
C3	0.0224 (7)	0.0430 (8)	0.0256 (7)	0.0025 (6)	0.0001 (5)	−0.0057 (6)
C18	0.0343 (7)	0.0289 (7)	0.0266 (7)	0.0014 (6)	0.0148 (6)	−0.0016 (5)
C20	0.0221 (7)	0.0307 (7)	0.0331 (7)	0.0046 (5)	0.0025 (5)	0.0019 (6)
C1	0.0384 (8)	0.0276 (7)	0.0301 (7)	−0.0034 (6)	0.0077 (6)	−0.0012 (6)
C31	0.0397 (8)	0.0232 (7)	0.0420 (8)	−0.0045 (6)	0.0206 (7)	−0.0024 (6)
C32	0.0438 (9)	0.0266 (7)	0.0615 (11)	−0.0123 (7)	0.0304 (8)	−0.0067 (7)
C34	0.0244 (7)	0.0379 (8)	0.0513 (9)	−0.0051 (6)	0.0086 (6)	−0.0074 (7)
C33	0.0283 (8)	0.0346 (8)	0.0708 (12)	−0.0142 (6)	0.0214 (8)	−0.0154 (8)

### Geometric parameters (Å, º)

**Table:**

O1—C9	1.3792 (14)	C15—C14	1.3768 (18)
O1—C13	1.3747 (14)	C26—H26	0.9500
O2—C28	1.2246 (15)	C26—C25	1.3890 (19)
O3—C35	1.3558 (17)	C14—H14	0.9500
O3—H3	1.02 (3)	C25—H25	0.9500
N3—N4	1.3769 (14)	C29—H29	0.9500
N3—C28	1.3833 (15)	C29—C30	1.4561 (19)
N3—C11	1.4982 (14)	C21—H21A	0.9900
N2—C16	1.3758 (16)	C21—H21B	0.9900
N2—C21	1.4599 (16)	C21—C20	1.5216 (19)
N2—C19	1.4572 (16)	C30—C35	1.407 (2)
N4—C29	1.2870 (17)	C30—C31	1.4025 (19)
N1—C5	1.3834 (16)	C4—H4A	0.9900
N1—C4	1.4569 (17)	C4—H4B	0.9900
N1—C2	1.4551 (16)	C4—C3	1.525 (2)
C27—C22	1.3864 (17)	C19—H19A	0.9900
C27—C28	1.4803 (17)	C19—H19B	0.9900
C27—C26	1.3907 (17)	C19—C18	1.5227 (18)
C12—C13	1.3879 (16)	C2—H2A	0.9900
C12—C11	1.5113 (16)	C2—H2B	0.9900
C12—C14	1.3970 (17)	C2—C1	1.5183 (19)
C9—C8	1.3892 (17)	C35—C34	1.400 (2)
C9—C10	1.3861 (17)	C3—H3A	0.9800
C22—C11	1.5215 (16)	C3—H3B	0.9800
C22—C23	1.3885 (17)	C3—H3C	0.9800
C8—C11	1.5163 (16)	C18—H18A	0.9800
C8—C7	1.3990 (17)	C18—H18B	0.9800
C13—C17	1.3878 (17)	C18—H18C	0.9800
C17—H17	0.9500	C20—H20A	0.9800
C17—C16	1.3997 (17)	C20—H20B	0.9800
C16—C15	1.4172 (17)	C20—H20C	0.9800
C10—H10	0.9500	C1—H1A	0.9800
C10—C5	1.3996 (17)	C1—H1B	0.9800
C7—H7	0.9500	C1—H1C	0.9800
C7—C6	1.3798 (18)	C31—H31	0.9500
C5—C6	1.4165 (18)	C31—C32	1.380 (2)
C6—H6	0.9500	C32—H32	0.9500
C24—H24	0.9500	C32—C33	1.385 (3)
C24—C23	1.3949 (17)	C34—H34	0.9500
C24—C25	1.3941 (19)	C34—C33	1.379 (2)
C23—H23	0.9500	C33—H33	0.9500
C15—H15	0.9500		
			
C13—O1—C9	118.45 (9)	C24—C25—H25	119.5
C35—O3—H3	107.4 (13)	C26—C25—C24	120.91 (11)
N4—N3—C28	128.56 (10)	C26—C25—H25	119.5
N4—N3—C11	115.17 (9)	N4—C29—H29	120.1
C28—N3—C11	114.74 (9)	N4—C29—C30	119.81 (12)
C16—N2—C21	120.92 (10)	C30—C29—H29	120.1
C16—N2—C19	120.53 (10)	N2—C21—H21A	108.5
C19—N2—C21	118.42 (10)	N2—C21—H21B	108.5
C29—N4—N3	120.51 (11)	N2—C21—C20	114.97 (11)
C5—N1—C4	119.81 (10)	H21A—C21—H21B	107.5
C5—N1—C2	121.79 (11)	C20—C21—H21A	108.5
C2—N1—C4	117.32 (10)	C20—C21—H21B	108.5
C22—C27—C28	109.72 (10)	C35—C30—C29	122.63 (12)
C22—C27—C26	121.67 (12)	C31—C30—C29	118.74 (13)
C26—C27—C28	128.59 (11)	C31—C30—C35	118.62 (13)
C13—C12—C11	122.36 (10)	N1—C4—H4A	108.9
C13—C12—C14	115.90 (11)	N1—C4—H4B	108.9
C14—C12—C11	121.66 (10)	N1—C4—C3	113.50 (11)
O1—C9—C8	123.28 (10)	H4A—C4—H4B	107.7
O1—C9—C10	114.06 (10)	C3—C4—H4A	108.9
C10—C9—C8	122.65 (11)	C3—C4—H4B	108.9
C27—C22—C11	110.76 (10)	N2—C19—H19A	108.7
C27—C22—C23	120.92 (11)	N2—C19—H19B	108.7
C23—C22—C11	128.22 (11)	N2—C19—C18	114.24 (11)
C9—C8—C11	122.00 (11)	H19A—C19—H19B	107.6
C9—C8—C7	116.08 (11)	C18—C19—H19A	108.7
C7—C8—C11	121.92 (10)	C18—C19—H19B	108.7
O1—C13—C12	123.16 (11)	N1—C2—H2A	108.5
O1—C13—C17	114.14 (10)	N1—C2—H2B	108.5
C12—C13—C17	122.71 (11)	N1—C2—C1	115.10 (11)
C13—C17—H17	119.5	H2A—C2—H2B	107.5
C13—C17—C16	121.01 (11)	C1—C2—H2A	108.5
C16—C17—H17	119.5	C1—C2—H2B	108.5
O2—C28—N3	126.36 (12)	O3—C35—C30	122.46 (12)
O2—C28—C27	128.73 (11)	O3—C35—C34	117.59 (14)
N3—C28—C27	104.91 (10)	C34—C35—C30	119.94 (14)
N2—C16—C17	121.29 (11)	C4—C3—H3A	109.5
N2—C16—C15	121.91 (11)	C4—C3—H3B	109.5
C17—C16—C15	116.80 (11)	C4—C3—H3C	109.5
C9—C10—H10	119.6	H3A—C3—H3B	109.5
C9—C10—C5	120.88 (11)	H3A—C3—H3C	109.5
C5—C10—H10	119.6	H3B—C3—H3C	109.5
N3—C11—C12	109.86 (9)	C19—C18—H18A	109.5
N3—C11—C22	99.47 (9)	C19—C18—H18B	109.5
N3—C11—C8	110.89 (9)	C19—C18—H18C	109.5
C12—C11—C22	114.63 (10)	H18A—C18—H18B	109.5
C12—C11—C8	110.56 (9)	H18A—C18—H18C	109.5
C8—C11—C22	110.96 (9)	H18B—C18—H18C	109.5
C8—C7—H7	118.6	C21—C20—H20A	109.5
C6—C7—C8	122.85 (11)	C21—C20—H20B	109.5
C6—C7—H7	118.6	C21—C20—H20C	109.5
N1—C5—C10	120.35 (11)	H20A—C20—H20B	109.5
N1—C5—C6	122.44 (11)	H20A—C20—H20C	109.5
C10—C5—C6	117.20 (11)	H20B—C20—H20C	109.5
C7—C6—C5	120.31 (11)	C2—C1—H1A	109.5
C7—C6—H6	119.8	C2—C1—H1B	109.5
C5—C6—H6	119.8	C2—C1—H1C	109.5
C23—C24—H24	119.4	H1A—C1—H1B	109.5
C25—C24—H24	119.4	H1A—C1—H1C	109.5
C25—C24—C23	121.21 (12)	H1B—C1—H1C	109.5
C22—C23—C24	117.65 (11)	C30—C31—H31	119.5
C22—C23—H23	121.2	C32—C31—C30	120.98 (16)
C24—C23—H23	121.2	C32—C31—H31	119.5
C16—C15—H15	119.7	C31—C32—H32	120.1
C14—C15—C16	120.59 (11)	C31—C32—C33	119.70 (15)
C14—C15—H15	119.7	C33—C32—H32	120.1
C27—C26—H26	121.2	C35—C34—H34	120.1
C25—C26—C27	117.57 (12)	C33—C34—C35	119.83 (16)
C25—C26—H26	121.2	C33—C34—H34	120.1
C12—C14—H14	118.5	C32—C33—H33	119.5
C15—C14—C12	122.99 (11)	C34—C33—C32	120.91 (14)
C15—C14—H14	118.5	C34—C33—H33	119.5
			
O1—C9—C8—C11	0.75 (17)	C10—C9—C8—C11	−178.13 (11)
O1—C9—C8—C7	179.83 (10)	C10—C9—C8—C7	0.94 (17)
O1—C9—C10—C5	−178.87 (10)	C10—C5—C6—C7	2.22 (18)
O1—C13—C17—C16	179.34 (10)	C11—N3—N4—C29	168.12 (11)
O3—C35—C34—C33	179.94 (14)	C11—N3—C28—O2	175.66 (11)
N3—N4—C29—C30	175.92 (11)	C11—N3—C28—C27	−4.11 (13)
N2—C16—C15—C14	179.21 (11)	C11—C12—C13—O1	3.95 (18)
N4—N3—C28—O2	10.6 (2)	C11—C12—C13—C17	−176.23 (11)
N4—N3—C28—C27	−169.16 (11)	C11—C12—C14—C15	176.21 (11)
N4—N3—C11—C12	−66.11 (12)	C11—C22—C23—C24	173.34 (11)
N4—N3—C11—C22	173.25 (9)	C11—C8—C7—C6	178.70 (11)
N4—N3—C11—C8	56.40 (13)	C7—C8—C11—N3	56.81 (14)
N4—C29—C30—C35	−3.20 (19)	C7—C8—C11—C12	178.91 (10)
N4—C29—C30—C31	178.18 (12)	C7—C8—C11—C22	−52.75 (15)
N1—C5—C6—C7	−176.72 (12)	C5—N1—C4—C3	65.09 (16)
C27—C22—C11—N3	−5.88 (12)	C5—N1—C2—C1	−86.26 (15)
C27—C22—C11—C12	−122.98 (11)	C23—C22—C11—N3	177.66 (11)
C27—C22—C11—C8	110.92 (11)	C23—C22—C11—C12	60.56 (16)
C27—C22—C23—C24	−2.80 (17)	C23—C22—C11—C8	−65.54 (15)
C27—C26—C25—C24	−1.80 (18)	C23—C24—C25—C26	1.20 (19)
C12—C13—C17—C16	−0.50 (18)	C26—C27—C22—C11	−174.51 (11)
C9—O1—C13—C12	−5.36 (16)	C26—C27—C22—C23	2.25 (18)
C9—O1—C13—C17	174.80 (10)	C26—C27—C28—O2	−1.4 (2)
C9—C8—C11—N3	−124.17 (12)	C26—C27—C28—N3	178.35 (12)
C9—C8—C11—C12	−2.06 (15)	C14—C12—C13—O1	−179.16 (11)
C9—C8—C11—C22	126.28 (12)	C14—C12—C13—C17	0.67 (17)
C9—C8—C7—C6	−0.38 (18)	C14—C12—C11—N3	−54.17 (14)
C9—C10—C5—N1	177.28 (11)	C14—C12—C11—C22	56.81 (15)
C9—C10—C5—C6	−1.68 (17)	C14—C12—C11—C8	−176.88 (10)
C22—C27—C28—O2	−179.82 (12)	C25—C24—C23—C22	1.12 (18)
C22—C27—C28—N3	−0.05 (13)	C29—C30—C35—O3	1.0 (2)
C22—C27—C26—C25	0.11 (18)	C29—C30—C35—C34	−178.41 (13)
C8—C9—C10—C5	0.11 (18)	C29—C30—C31—C32	179.28 (13)
C8—C7—C6—C5	−1.23 (19)	C21—N2—C16—C17	0.60 (17)
C13—O1—C9—C8	2.99 (16)	C21—N2—C16—C15	−178.94 (11)
C13—O1—C9—C10	−178.03 (10)	C21—N2—C19—C18	−102.27 (13)
C13—C12—C11—N3	122.54 (12)	C30—C35—C34—C33	−0.6 (2)
C13—C12—C11—C22	−126.47 (12)	C30—C31—C32—C33	−1.0 (2)
C13—C12—C11—C8	−0.16 (15)	C4—N1—C5—C10	20.76 (18)
C13—C12—C14—C15	−0.71 (18)	C4—N1—C5—C6	−160.34 (12)
C13—C17—C16—N2	−179.25 (11)	C4—N1—C2—C1	81.84 (15)
C13—C17—C16—C15	0.31 (17)	C19—N2—C16—C17	−175.33 (11)
C17—C16—C15—C14	−0.34 (18)	C19—N2—C16—C15	5.13 (17)
C28—N3—N4—C29	−26.88 (18)	C19—N2—C21—C20	96.50 (13)
C28—N3—C11—C12	126.76 (11)	C2—N1—C5—C10	−171.43 (11)
C28—N3—C11—C22	6.13 (12)	C2—N1—C5—C6	7.48 (18)
C28—N3—C11—C8	−110.73 (11)	C2—N1—C4—C3	−103.26 (13)
C28—C27—C22—C11	4.02 (13)	C35—C30—C31—C32	0.6 (2)
C28—C27—C22—C23	−179.22 (10)	C35—C34—C33—C32	0.2 (2)
C28—C27—C26—C25	−178.12 (12)	C31—C30—C35—O3	179.64 (12)
C16—N2—C21—C20	−79.52 (15)	C31—C30—C35—C34	0.2 (2)
C16—N2—C19—C18	73.76 (15)	C31—C32—C33—C34	0.6 (2)
C16—C15—C14—C12	0.57 (19)		

### Hydrogen-bond geometry (Å, º)

*Cg* is the centroid of the C22–C27 ring.

**Table:**

*D*—H···*A*	*D*—H	H···*A*	*D*···*A*	*D*—H···*A*
C31—H31···O3^i^	0.95	2.77	3.4737 (19)	131
O3—H3···N4	1.02 (3)	1.72 (2)	2.6276 (15)	145 (2)
C18—H18*A*···*Cg*^ii^	0.98	2.88	3.8191 (15)	161

Symmetry codes: (i) *x*, −*y*+3/2, *z*−1/2; (ii) −*x*+1, −*y*+1, −*z*.
